# Cardiovascular magnetic resonance evaluation of left ventricular peak filling rate using steady-state free precession and phase contrast sequences

**DOI:** 10.1186/s40064-016-2878-x

**Published:** 2016-07-25

**Authors:** Shotaro Komi, Yusuke Inoue, Hirofumi Hata, Ai Nakajima, Hiroki Miyatake

**Affiliations:** 1 Department of Radiology, Kitasato University Hospital, Sagamihara, Kanagawa Japan; 2 Department of Diagnostic Radiology, Kitasato University School of Medicine, 1-15-1 Kitasato, Minami-ku, Sagamihara, Kanagawa 252-0374 Japan

**Keywords:** Cardiovascular magnetic resonance, Diastolic function, Peak filling rate, Phase contrast, Steady-state free precession

## Abstract

**Background:**

We investigated a practical method to measure peak filling rate (PFR) as an indicator of diastolic function of the left ventricle. Ten adult volunteers underwent cine MR imaging using steady-state free precession (SSFP) and phase contrast (PC) sequences to measure PFR. Two PC image sets were acquired at the mitral valve orifice, and PFR was determined from the set with high true temporal resolution (temporal PC method) or with high spatial resolution (spatial PC method). SSFP images covering the left ventricle were acquired, and a time–volume curve was generated around the peak filling phase. PFR was determined using parabolic curve fitting on the first-derivative curve of the LV time–volume curve.

**Findings:**

PFR values estimated by the PC methods correlated well with those estimated by the SSFP method, despite apparent underestimation. The underestimation was smaller for the temporal PC method (12 %) than for the spatial PC method (28 %). Intra- and inter-observer repeatabilities were better for the PC methods than for the SSFP method.

**Conclusions:**

PFR measurement by PC imaging with high true temporal resolution is convenient and offers excellent repeatability and acceptable accuracy, indicating suitability for clinical use.

## Background

Diastolic function of the left ventricle (LV) is often impaired before development of systolic dysfunction (Mandinov et al. 2000), and is commonly evaluated by echocardiography (Yamada and Klein 2010; Kasner et al. 2007; Oh et al. 2006). Cine cardiovascular magnetic resonance (CMR) imaging using a steady-state free precession (SSFP) sequence has been established as a reliable method for the measurement of LV volume and systolic function (Ichikawa et al. 2003; Attili et al. 2010; Finn et al. 2006). Although the peak filling rate (PFR) can be also calculated as an indicator of diastolic function, the image analysis takes a long time because of the need for manual demarcation of the LV cavity on many slices, preventing widespread use of this method (Leong et al. 2010). Phase contrast (PC) cine CMR imaging can provide PFR values by measurement of mitral flow volumes (Rubinshtein et al. 2009; Beeres et al. 2008; Ashrafpoor et al. 2015). Manual tracing of the contour is required only on one slice at peak filling, and the convenience may make this method suitable for clinical use.

In the present study, we measured PFR in adult volunteers using SSFP and PC sequences to validate PFR measurement by PC imaging regarding that by SSFP imaging as a standard. High temporal resolution is essential for SSFP CMR assessment of PFR (Miller et al. 2002), which is assumed to hold true for PC CMR assessment. However, higher temporal resolution requires lower spatial resolution to avoid prolongation of scan time. We compared the effects of temporal resolution and spatial resolution on PFR measurement. In addition, we compared intra- and inter-observer variabilities between SSFP and PC methods. The aim of this study was to determine a practical CMR method to assess PFR.

## Methods

### Subjects

The study subjects comprised ten adult volunteers (seven males and three females) with no history of cardiac or chronic diseases and no contraindications to CMR imaging. The age was 30.5 ± 6.2 (mean ± SD) years, height was 169.5 ± 10.5 cm, and weight was 60.2 ± 12.8 kg. The heart rate, systolic blood pressure, and diastolic blood pressure were 69.1 ± 6.6 bpm, 125.4 ± 15.3, and 70.9 ± 10.6 mmHg, respectively. The Institutional Review Board for Observation and Epidemiological Study, Kitasato University Medical Ethics Organization approved the study (KMEO B13-89), and all participants provided written informed consent.

### Imaging procedures

All CMR studies were performed on a 1.5 T clinical scanner (Signa HDxt; GE Healthcare, Milwaukee, WI, USA) with an eight-channel phased-array coil. All images were obtained during breath holding at expiration. After localizing scans, vertical and horizontal long-axis images were obtained using an SSFP cine sequence.

On end-systolic images of both long-axis directions, one slice was planned for PC cine imaging at the position of the mitral valve orifice and in parallel to it. Two image sets, one with high true temporal resolution (temporal PC) and one with high spatial resolution (spatial PC), were acquired using different views per segment (VPS) and different acquisition matrix sizes (Table 1). The reconstruction matrix (256 × 256) and reconstruction pixel size (1.2 × 1.2 mm) were identical between both sets. Images at 64 cardiac phases were reconstructed using retrospective gating. Although the apparent temporal resolution, defined as the interval between adjacent reconstructed phases, was identical between the two sets and ranged from 13.8 to 17.0 ms, depending on the heart rate, true temporal resolution, defined as TR × VPS × 2 in PC cine imaging (Foo et al. 1995), was better for temporal PC imaging. One-dimensional velocity encoding was applied perpendicularly to the slice plane, and the velocity sensitivity threshold was set at 150 cm/s. Other scan parameters were as follows: flip angle, 20°; number of excitations (NEX), 1; FOV, 300 × 300 mm; slice thickness, 5 mm; acquisition time, 14 s at a heart rate of 60 bpm. A parallel imaging technique [array spatial sensitivity encoding technique (ASSET)] was used with a reduction factor of 2.

**Table 1:** Imaging parameters for PC cine MRI

Parameters	Temporal PC	Spatial PC
TR (ms)	5.4–5.5	6.3–6.4
TE (ms)	3.6–3.7	3.8–3.9
VPS	4	8
Acquisition matrix	96 × 96	256 × 192
Acquisition pixel size (mm)	3.1 × 3.1	1.2 × 1.6
True temporal resolution (ms)	43.2–44.0	100.8–102.4

Contiguous short-axis images covering the entire LV were planned for SSFP cine imaging on end-diastolic SSFP images of both long-axis directions. Images at 32 cardiac phases were reconstructed using retrospective gating. The scan parameters were as follows: TR, 4.0 ms; TE, 1.7 ms; flip angle, 50°; NEX, 0.5; FOV, 340 × 340 mm; slice thickness, 8 mm; slice gap, 0 mm; acquisition matrix, 224 × 224; VPS, 10. True temporal resolution, defined as TR × VPS in SSFP cine imaging, ranged from 39 to 40 ms, and apparent temporal resolution varied from 27.6 to 34.1 ms, depending on the heart rate. ASSET was used with a reduction factor of 2, and the acquisition time was 9 s/slice at a heart rate of 60 bpm.

### Determination of PFR by PC imaging

**Fig. 1:**
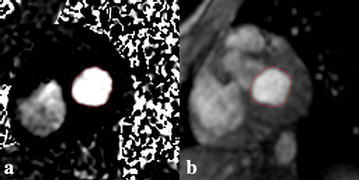
Examples of the phase (**a**) and magnitude (**b**) images of PC cine imaging. *Red lines* indicate the contour of mitral flow traced manually

Dedicated software, cvi^42^ (Circle Cardiovascular Imaging Inc., Calgary, AB, Canada), was used for image analysis. On the phase images of PC imaging, an operator visually determined the peak filling phase during early filling. The region corresponding to the LV inflow through the mitral valve orifice was manually demarcated on the phase image at peak filling, referring to the magnitude image of PC imaging (Fig. 1). On phase images, the positivity or negativity of a pixel value represents the flow direction at the pixel. When the mitral flow produced positive pixel values, the operator set the upper and lower limits of the display window at 400 and 0, respectively (i.e., window width, 400; window level, 200), in manual demarcation. In the case of negative values, the upper and lower limits of the display window were set at 0 and −400, respectively (window width, 400; window level, −200), and the gray scale was reversed. The mitral flow volume (mL/s) was estimated as the area multiplied by the absolute value of the mean signal intensity representing mean velocity. Considering possible inaccuracy of the visual determination of peak filling, the flow volume was also estimated at one cardiac phase each immediately before and immediately after the visual peak filling phase. PFR was defined as the maximum value among the flow volumes estimated at the three consecutive phases and was calculated from temporal PC and spatial PC images independently. Peak filling time was also recorded.

### Determination of PFR by SSFP imaging

**Fig. 2:**
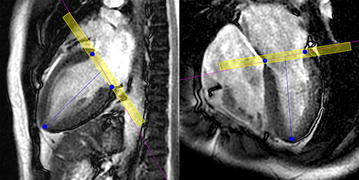
Use of the long-axis views for LV volumetry from SSFP imaging. The operator manually specified the basal end and apex of the left ventricle (*blue dots*) on the *vertical* (*left*) and *horizontal* (*right*) long-axis views. The basal slice contained the left atrium and LV even within a pixel, and the processing using the long-axis images aided extraction of the true LV volume. The *yellow box*, representing the volume of the basal slice, was added for explanation to the actual display of the cvi^42^ software

The peak filling phase during early filling was determined visually on the temporal PC images, and LV volumes were calculated using cvi^42^ from SSFP images obtained at five or six consecutive phases centered at the visual peak filling phase. Images were reconstructed at 32 and 64 cardiac phases in SSFP and PC images, respectively, and ideally the timing was identical between the n phase (n = natural number) in SSFP imaging and the (2n − 1) phase in PC imaging. When the peak filling appeared at the odd-numbered phase (2n − 1) in temporal PC imaging, five phases (n − 2, n − 1, n, n + 1, n + 2) were used for the analysis of SSFP images. When the peak filling appeared at the even-numbered phase (2n) in temporal PC imaging, the peak corresponded to the center between the n and (n + 1) phases in SSFP imaging and six phases (n − 2, n − 1, n, n + 1, n + 2, n + 3) were used. At the phases for analysis, endocardial contours were traced manually on all short-axis images encompassing the LV cavity, and the LV volume was calculated. Papillary muscles were assigned to the LV cavity. On the basal slice including the LV outflow tract, the outflow tract and LV were divided by a straight line. The operator manually specified the basal end and apex of the LV on the vertical and horizontal long-axis views at each phase (Fig. 2), and the short-axis slices encompassing the LV were selected automatically. The partial volume effect inevitably caused mixing of the left atrium and LV within a pixel in the basal slice, and the software allowed separating the left atrium and LV within the basal slice through this processing on the long-axis images.

The first-derivative curve of the LV time–volume curve was generated by plotting the change rates of the LV volume between two consecutive phases (mL/s) against time, and a parabolic curve was fitted to the first-derivative curve to preserve temporal resolution while reducing the effect of erroneous variation related to observer-dependent contour demarcation. PFR and peak filling time were determined from the peak of the parabolic curve. Additionally, the end-diastolic volume (EDV), end-systolic volume (ESV), EF, and LV mass at end diastole were calculated from SSFP images. EDV, ESV, and mass were indexed to body surface area (BSA).

### Data analysis

The relationship between PFRs estimated by two methods was assessed by linear regression analysis and Bland–Altman analysis. To evaluate intra- and inter-observer variabilities in estimating PFR, one observer (S.K., 7 years of experience in CMR imaging) repeated the analysis with an interval of at least 2 weeks, and another observer (A.N., 4 years of experience in CMR imaging) analyzed the data independently. The intra- and inter-observer variabilities were assessed by Bland–Altman analysis.

### Statistical analysis

Data are expressed as mean ± SD. Linear regression analysis was performed by the least squares method. Peak filling times obtained by three methods were compared using one-way analysis of variance. A *P* value <0.05 was deemed statistically significant. The intraclass correlation coefficient (ICC) was calculated to evaluate inter- and intra-observer repeatabilities. The Statistical Package for Social Sciences (SPSS) version 21.0 for Windows (SPSS Inc., Chicago, IL, USA) was used.

## Results

Complete sets of SSFP and PC cine images were acquired in all ten subjects. The heart rate was 62.1 ± 5.2 bpm upon examination. EDV, ESV, EF, and mass were 149.5 ± 25.9 mL (range 101.4–176.7 mL), 65.1 ± 14.1 mL (range 40.2–81.1 mL), 56.7 ± 3.1 % (range 52.0–62.7 %), and 94.9 ± 25.6 g (range 52.1–129.2 g), respectively. EDV/BSA, ESV/BSA, and mass/BSA were 88.8 ± 8.0 mL/m^2^ (range 76.8–99.8 mL/m^2^), 38.5 ± 5.2 mL/m^2^ (range 29.3–46.5 mL/m^2^), and 55.7 ± 9.4 g/m^2^ (range 39.4–68.2 g/m^2^), respectively.

**Fig. 3:**
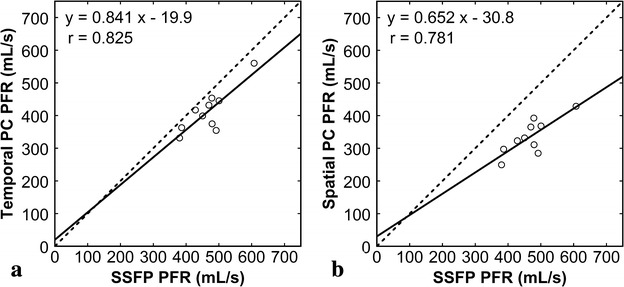
Comparison of PFRs estimated by various methods. PFR values estimated by the temporal PC method (**a**) and spatial PC method (**b**) were plotted against those estimated by the SSFP method. The *solid lines* represent the regression lines, and the *broken lines* represent the line of identity

**Fig. 4:**
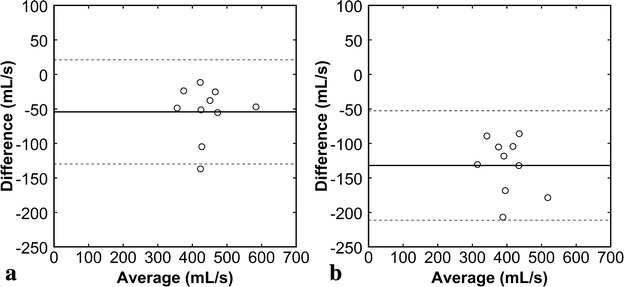
Bland–Altman plots for PFRs estimated by various methods. The temporal PC method (**a**) and spatial PC method (**b**) were compared with the SSFP method. The difference between two methods was plotted against their average. The *solid line* represents the mean of the differences, and the *broken lines* represent the mean ± 1.96 SD (95 % CI)

PFR was estimated as 467.3 ± 64.5, 413.1 ± 65.8, and 335.4 ± 53.9 mL/s by the SSFP, temporal PC, and spatial PC methods, respectively. PFR values estimated by the temporal PC [r = 0.825, 95 % confidence interval (CI) 0.408–0.957, Fig. 3a] and spatial PC (r = 0.781, 95 % CI 0.299–0.946, Fig. 3b) methods correlated well with those estimated by the SSFP method. Mean PFR estimated by the temporal PC method was 12 % smaller than that estimated by the SSFP method. The bias was −54.3 ± 38.5 mL/s (95 % CI −81.3 to −26.7 mL/s), the lower limits of agreement was −129.8 mL (95 % CI −177.5 to −82 mL/s), and the upper limits of agreement was 21.2 mL/s (95 % CI −26.5 to 69.0 mL/s) (Fig. 4a). Mean PFR estimated by the spatial PC method was 28 % smaller than that estimated by the SSFP method. The bias was −132.0 ± 40.4 mL/s (95 % CI −160.9 to −103.1 mL/s), the lower limits of agreement was −211.2 mL/s (95 % CI −261.3 to −161.1 mL/s), and the upper limits of agreement was −52.7 mL/s (95 % CI −102.8 to −2.6 mL/s) (Fig. 4b). Peak filling time was estimated as 457.5 ± 31.9 s, 466.2 ± 18.2 s, and 475.6 ± 23.9 s by the SSFP, temporal PC, and spatial PC methods, respectively, showing no significant difference (*P* = 0.294).

**Fig. 5:**
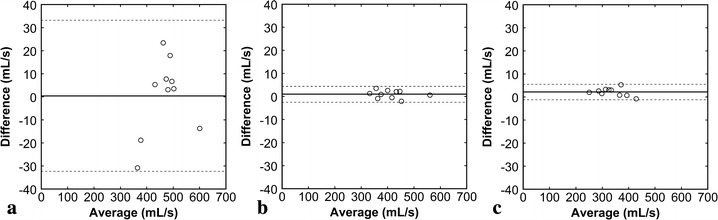
Bland–Altman plots representing intraobserver variabilities (**a** SSFP method, **b** temporal PC method, **c** spatial PC method). The difference between the two estimates by an observer was plotted against their average. The *solid line* represents the mean of the differences, and the *broken lines* represent the mean ± 1.96 SD (95 % CI)

**Fig. 6:**
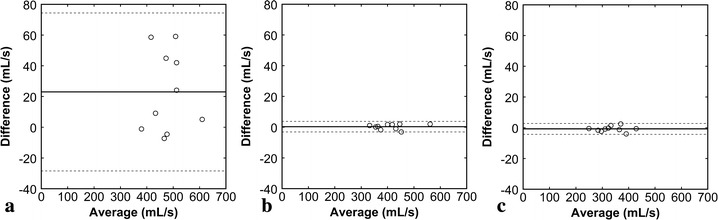
Bland–Altman plots representing interobserver variabilities (**a** SSFP method, **b** temporal PC method, **c** spatial PC method). The difference between the two observers were plotted against their average. The *solid line* represents the mean of the differences, and the *broken lines* represent the mean ± 1.96 SD (95 % CI)

Intraobserver comparisons demonstrated that the biases were close to zero for all methods, indicating limited systematic errors (Fig. 5). However, the 95 % CIs were definitely wider and ICCs smaller (Table 2) for the SSFP method than for the PC methods, indicating larger random errors for the SSFP method. Interobserver comparisons revealed that both systematic and random errors were larger for the SSFP method than for the PC methods (Fig. 6; Table 2).

**Table 2:** ICC for intra- and inter-observer variability

Methods	Intraobserver	Interobserver
SSFP	0.972	0.871
Temporal PC	1.000	1.000
Spatial PC	0.999	0.999

## Discussion

To estimate PFR using SSFP cine CMR imaging, the LV contour is demarcated on many slices at many cardiac phases, which takes a long time. Because even minor fluctuation of the LV volume may cause a large error in the change rate of the volume (Theisen et al. 2013), the observer should be particularly careful in the demarcation. The considerable burden on observers has limited its widespread use. In the present study, manual demarcation was performed only around the peak filling phase determined visually on PC images, which reduced the observer’s burden.

PC cine CMR imaging at the mitral valve orifice allow to estimate PFR via measurement of the LV inflow over time (Rubinshtein et al. 2009; Beeres et al. 2008; Ashrafpoor et al. 2015). This estimation requires data acquisition of only one slice and image analysis of only one cardiac phase, offering substantial convenience. The present study demonstrated that PFR estimated by the PC method correlated well with that estimated by the SSFP method, despite apparent underestimation. Although more sophisticated techniques such valve tracking may further improve accuracy (Brandts et al. 2011), the simple PFR measurement using PC cine imaging appears to be a practical and widely available method for evaluation of diastolic function with an acceptable burden on the patient and operator.

PFR estimates were compared between PC imaging with high true temporal resolution and that with high spatial resolution. PFR was systematically underestimated for both sets compared with PFR estimated by the SSFP method; however, the degree of underestimation was smaller using high true temporal resolution. It is suggested that true temporal resolution is more important than spatial resolution for measuring PFR by PC imaging. Improvement of true temporal resolution prolongs acquisition time and, consequently, breath-holding time. Reducing spatial resolution appears to be acceptable to prevent excessive prolongation of the acquisition time. In SSFP imaging, the influence of true temporal resolution is greater for PFR measurement than for EF measurement (Miller et al. 2002). It is suggested that PC imaging with low temporal resolution also underestimates PFR due to rapid changes in the filling rate. Apparent temporal resolution, defined as the interval between the adjacent reconstructed phases, was identical for the two sets of PC images with high true temporal resolution and high spatial resolution. In SSFP imaging, a reduction in true temporal resolution has been indicated to cause underestimation of EF even with high apparent temporal resolution (Inoue et al. 2005). The results of the present study have suggested that improvement of the apparent temporal resolution does not effectively compensate for low true temporal resolution in the measurement of PFR by PC imaging.

PFR values estimated from a given set of images may vary depending on observer-dependent demarcation of the LV contour. In the present study, better inter- and intraobserver repeatabilities in PFR measurement were demonstrated for the PC methods compared with the SSFP method. The SSFP method produced larger random errors in intraobserver comparison and larger systematic and random errors in interobserver comparison compared with the PC methods. The area of the demarcated LV region directly affects the estimation of PFR from SSFP images. By contrast, PFR measurement from PC images utilizes the total signal in the demarcated region and is less susceptible to observer-dependent contour demarcation than measurement from SSFP images due to low signal intensity near the contour. The excellent inter- and intraobserver repeatabilities support the clinical usefulness of PFR measurement by PC imaging.

In the present study, only a small number of adult volunteers were examined using CMR imaging alone. Because echocardiography is a current standard for the evaluation of diastolic function, comparison of the CMR method with echocardiographic method in a large number of patients with LV dysfunction should be conducted in the future.

## Conclusion

We investigated methods of measuring PFR using CMR imaging. PFR values estimated by PC imaging correlated well with those estimated by SSFP imaging, despite systematic underestimation. In the measurement of PFR by PC imaging, true temporal resolution was more important than spatial resolution, and improvement in the apparent temporal resolution did not effectively compensate for low true temporal resolution. Inter- and intra-observer repeatabilities were better for the PC methods than for the SSFP methods. PFR measurement by PC imaging with high temporal resolution is convenient and offers excellent repeatability and acceptable accuracy, suggesting its suitability for clinical use as an adjunct to CMR assessment of systolic function.

## Abbreviations

CI: confidence intervals
ICC: intraclass correlation coefficient
PC: phase contrast
PFR: peak filling rate
SSFP: steady-state free precession
VPS: views per segment

## References

[CR1] Ashrafpoor G, Bollache E, Redheuil A, De Cesare A, Giron A, Defrance C, Azarine A, Perdrix L, Ladouceur M, Diebold B, Mousseaux E, Kachenoura N (2015). Age-specific changes in left ventricular diastolic function: a velocity-encoded magnetic resonance imaging study. Eur Radiol.

[CR2] Attili AK, Schuster A, Nagel E, Reiber JH, van der Geest RJ (2010). Quantification in cardiac MRI: advances in image acquisition and processing. Int J Cardiovasc Imaging.

[CR3] Beeres SL, Lamb HJ, Roes SD, Holman ER, Kaandorp TA, Fibbe WE, de Roos A, van der Wall EE, Schalij MJ, Bax JJ, Atsma DE (2008). Effect of intramyocardial bone marrow cell injection on diastolic function in patients with chronic myocardial ischemia. J Magn Reson Imaging.

[CR4] Brandts A, Bertini M, van Dijk EJ, Delgado V, Marsan NA, van der Geest RJ, Siebelink HM, de Roos A, Bax JJ, Westenberg JJ (2011). Left ventricular diastolic function assessment from three-dimensional three-directional velocity-encoded MRI with retrospective valve tracking. J Magn Reson Imaging.

[CR5] Finn JP, Nael K, Deshpande V, Ratib O, Laub G (2006). Cardiac MR imaging: state of the technology. Radiology.

[CR6] Foo TK, Bernstein MA, Aisen AM, Hernandez RJ, Collick BD, Bernstein T (1995). Improved ejection fraction and flow velocity estimates with use of view sharing and uniform repetition time excitation with fast cardiac techniques. Radiology.

[CR7] Ichikawa Y, Sakuma H, Kitagawa K, Ishida N, Takeda K, Uemura S, Motoyasu M, Nakano T, Nozaki A (2003). Evaluation of left ventricular volumes and ejection fraction using fast steady-state cine MR imaging: comparison with left ventricular angiography. J Cardiovasc Magn Reson.

[CR8] Inoue Y, Nomura Y, Nakaoka T, Watanabe M, Kiryu S, Okubo T, Ohtomo K (2005). Effect of temporal resolution on the estimation of left ventricular function by cardiac MR imaging. Magn Reson Imaging.

[CR9] Kasner M, Westermann D, Steendijk P, Gaub R, Wilkenshoff U, Weitmann K, Hoffmann W, Poller W, Schultheiss HP, Pauschinger M, Tschöpe C (2007). Utility of Doppler echocardiography and tissue Doppler imaging in the estimation of diastolic function in heart failure with normal ejection fraction: a comparative Doppler-conductance catheterization study. Circulation.

[CR10] Leong DP, De Pasquale CG, Selvanayagam JB (2010). Heart failure with normal ejection fraction: the complementary roles of echocardiography and CMR imaging. JACC Cardiovasc Imaging.

[CR11] Mandinov L, Eberli FR, Seiler C, Hess OM (2000). Diastolic heart failure. Cardiovasc Res.

[CR12] Miller S, Simonetti OP, Carr J, Kramer U, Finn JP (2002). MR Imaging of the heart with cine true fast imaging with steady-state precession: influence of spatial and temporal resolutions on left ventricular functional parameters. Radiology.

[CR13] Oh JK, Hatle L, Tajik AJ, Little WC (2006). Diastolic heart failure can be diagnosed by comprehensive two-dimensional and Doppler echocardiography. J Am Coll Cardiol.

[CR14] Rubinshtein R, Glockner JF, Feng D, Araoz PA, Kirsch J, Syed IS, Oh JK (2009). Comparison of magnetic resonance imaging versus Doppler echocardiography for the evaluation of left ventricular diastolic function in patients with cardiac amyloidosis. Am J Cardiol.

[CR15] Theisen D, Sandner TA, Bamberg F, Bauner KU, Schwab F, Schwarz F, Arnoldi E, Reiser MF, Wintersperger BJ (2013). High-resolution cine MRI with TGRAPPA for fast assessment of left ventricular function at 3 Tesla. Eur J Radiol.

[CR16] Yamada H, Klein AL (2010). Diastology 2010: clinical approach to diastolic heart failure. J Echocardiogr.

